# Exploring Occupational Therapists’ Professional Identity: A Q-Method Study

**DOI:** 10.3390/healthcare11040630

**Published:** 2023-02-20

**Authors:** Ana-Isabel Souto-Gómez, Miguel-Ángel Talavera-Valverde, María-del-Pilar García-de-la-Torre, Luis-Javier Márquez-Álvarez

**Affiliations:** 1 Integra Saúde Unit Research, Escola Universitaria de Traballo Social, Universidade Santiago de Compostela, 15704 Santiago de Compostela, Spain; 2 Integra Saúde Unit Research, Health Science Department, Facultad de Ciencias de la Salud, Universidade da Coruña, 15570 A Coruña, Spain; 3 Área Sanitaria de Ferrol, 15405 Ferrol, Spain; 4 Psychology Department, Facultad de Ciencias de la Educación, Universidade da Coruña, 15008 A Coruña, Spain; 5 Área Sanitaria de Vigo, 36204 Vigo, Spain

**Keywords:** occupational therapy, occupational therapists, professional identity, professional, Q-method

## Abstract

(1) Background: This study examines the nature of the rarely studied factors of the professional identity from an occupational therapist’s perspective. (2) Methods: Q-methodology was applied to identify the different perspectives. Participants were selected through a non-probability sampling procedure in the whole Spanish territory. Different assessment tools were considered, in order to develop an ad hoc tool which had 40 statements classified into four categories. A factor analysis was performed by applying Ken-Q analysis v.1.0. (3) Results: Thirty-seven occupational therapists participated in the study. Their diverse approaches revealed different perspectives that influence the professional identity of occupational therapists: professional identity, due to referents, a grey field on professional identity, reaffirming a common professional identity, the role of education and mentors on professional identity and the outcome of ongoing training, in order to develop the abovementioned identity. (4) Conclusions: Once the different aspects of the professional identity have been understood, future educational initiatives can be designed to adapt curricula to the professional scene.

## 1. Introduction

Professional identity is a multidimensional concept that is constantly changing and evolving [1]. This process helps us to acquire knowledge, professional skills, an understanding of the reality, the ethical, and the professional requirements [2], as well as of personal and professional needs [3], along with the development of the moral context of the practice [4].

Research on professional identity in occupational therapy rarely focuses on professional identity aspects, values, environments, or beliefs prioritised and highlighted by occupational therapists. On the other hand, studies on the impact and development process of professional identity among students tend to have greater participation and, therefore, greater scientific popularity. Studies focused on professional identity in working professionals are in the minority [5,6,7,8].

Research on how professional identity is formed examines how early training experiences influence the professional identity of students of occupational therapy and how educational initiatives can be fostered to promote this identity [3,9,10,11,12]. Contributions from this line of work must not be ignored, but understanding the central question about what encompasses the professional identity of occupational therapists is relatively under-researched. 

Social constructivism [13,14], which has been extensively used in other disciplines, but only rarely applied to occupational therapy, states that identity encompasses a mutable process, that is to say, that emerges from a variety of values and beliefs issued from one’s environment, and it is transferred through language [15] and influenced, in turn, by values and beliefs.

Professional identity is also developed through interactions. Specific contexts or unique interpersonal dynamics may affect the expression of our own professional identity [14,16], and gaining experience is, thus, relevant, since identity may evolve depending on a variety of values and beliefs [17]. Therefore, we believe that professional identity gives sense to our profession [1].

Metaxas [18], Ikiugu, and Rosso [8] and Walder et al. [1] described professional identity in occupational therapy as formed through the process of identification: a two-way, internal and external, process that helps us define ourselves and is also defined by others. Professional identity encompasses complex and diverse components, such as personal background, fundamental values, and each one’s work culture. Organisational relations and working circumstances may equally become components that frame the development of professional identity, in order to develop professionalism in occupational therapy [19]. The factors that may contribute to the professional identity of occupational therapists are illustrative and non-exhaustive.

The study of professional identity in students and professionals is a topic explored in other disciplines, such as nursing [20,21], medicine [22,23], and physiotherapy [24,25]. Professional identity has also been studied within occupational therapy, as highlighted by Turner and Knight [26]. Currently, there are many occupational therapists who barely have professional identity, a situation that could be due to: (a) a confusion in their role as referred to by Guru et al. [27]; (b) the status of their professional identity described by Fanshawen [28], a problem also defined by Godber [29], who considered that occupational therapists lacked a unique identity.

This research is joined by others, such as Grant’s [30] in the UK, who studied the collective identity of the profession, identifying it as weak, or Zambonini’s [31], who studied how professional identity has evolved over 40 years in Brazil.

In contrast, in Spain, such research is not being conducted, which may have influenced the development of Spanish professional identity. As we have not found scientific evidence that analyses identity factors in occupational therapists in the Spanish context, we consider that this study presents an up-to-date topic.

This lack of research has led to an interest in delving into the analysis of the elements that influence professional identity throughout a professional’s career, an aspect not addressed in prior professional identity research. This research provides the necessary variables to reconstruct the curricula, in accordance with the occupational therapists’ professional identity practice, providing evidence to describe the curricular elements at a theoretical level that describe a practice dependent on an occupation paradigm [32].

Taking, as a starting point, the fact that professional identity is made up of different components, such as values, beliefs, and the environment, our research tries to find out which factors are used by professional therapists to describe their professional identity. Therefore, our survey includes the following question:

*RQ: What is the understanding that occupational therapists have of the factors that contribute to professional identity?*

Our research intends to examine the widespread beliefs that occupational therapists hold, regarding their professional identity, by applying the Q-methodology [33], which incorporates aspects of both quantitative and qualitative techniques to examine human subjectivity [34] within a rigorous and objective procedure [35].

## 2. Materials and Methods

### 2.1. Design

Q-methodology enables the subjectivity of participants to be preserved through an objective process, in which each participant provides his or her perspective by ordering different statements, according to a predetermined study question [36]. It has been used previously to study subjectivity on professional identity in health professions [37]. This tool enables a better understanding of people’s perspectives and beliefs, which are generated and explored through a specific method of data collection and statistical analysis [38]. It uses a specific statistical method that reduces a large number of variables into a smaller number of factors, in order to group people according to how they interpret statements about a topic [39]. Therefore, it is possible to identify participants’ viewpoints by requesting individuals to undertake an operant procedure (sorting related statements).

Our research complements a previous study performed by Márquez-Álvarez et al. [40], where the authors used the Q method to identify the perspective of occupational therapy students on concepts that were key to improving professional reasoning. This type of research is paramount to longitudinally study a population where professional reasoning leads to the construction of professional identity [41].

### 2.2. Development of the Q Sample

The Q sample is a set of statements representative of the majority of ideas present in the opinions of the field of study [36]. A concourse relating statements was generated, comprised of a collection of thoughts and information about professional identity. As relevant sources related to professional identity, different assessments and studies were gathered through a literature review [1,5,8,26,42,43]. An initial concourse of n = 53 statements was determined.

The statements were reviewed by three authors from the research team (AISG, MATV, and LJMA). In this case, initial statements were transcribed as personal statements from the selected scales in the development of the concourse, selecting the most representative ideas for study purposes. Each of the participants were selected separately the statements, and, in order to maintain the plurality of ideas for the analysis, duplicates were eliminated, and those with no consensus were included. Statements were subsequently refined to 43.

To enhance the rigour of the study, a Q-sort was piloted with a purposively selected sample of three occupational therapists, as Garbellini et al. [44] included in their method. Thanks to this, a further check of the comprehension and relevance of all the statements was considered. The final Q sample included n = 40 statements. For better legibility of the results, they were included in four different groups and coded as such (Table 1). There was no subsequent modification to this sample.

### 2.3. Selection of the P Set

The P set (set of participants) was assembled by non-probabilistic sampling for convenience. It comprised currently working professional therapists, who wished to take part in the study. Initial contact with professional associations and colleges was made for the recruitment. Participants were chosen based on their availability for the study, as well as their knowledge about professional identity. We included any occupational therapists who expressed interest on the theme and demonstrated basic understanding of the concept of professional identity. Additionally, we addressed any questions or concerns from participants to ensure a minimum level of understanding.

Given that the Q-methodology seeks to identify the different opinions within this group of participants, there is no need to make a minimum sample size calculation. A large sample is not required, as the aim of the Q method is to identify key opinions with the selected participant group. Per the literature, and following the indications of Watts and Stenner [45], an approximate 1:1 ratio of terms to people interviewed is required to conduct the study. The number of participants should not exceed the number of terms or phrases used. Therefore, professionals participated in the study until reached the specified number of n = 40. However, the final number of occupational therapists included in the study was n = 37, due to the withdrawal of three participants.

### 2.4. Q-Sort

**Figure 1:**
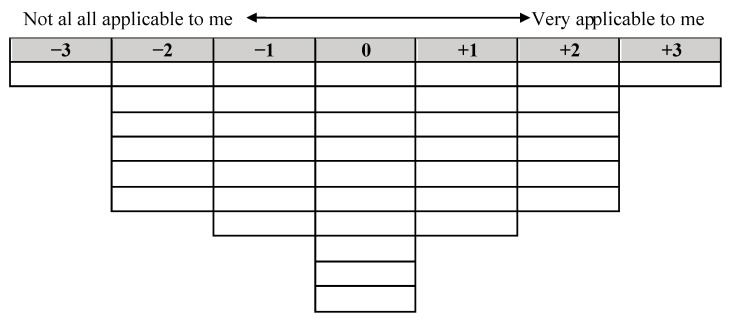
Representation of the grid for the Q-sort used in the online sorting procedure. Participants assigned all 40 statements on the places on the grid.

Before the participants were recruited, an initial design was proposed to the University of A Coruña’s Ethics Committee, with the approval number 2022-001. Following, in April and May 2022, an initial contact with professional colleges and associations of occupational therapist was established. Participants were recruited in May 2022, signed an informed consent for their participation, and ranked Q sample statements on a continuous scale, with a quasi-normal distribution between “most relevant” and “least relevant” (Figure 1). Each column had an added value, ranging from −3 to +3. Participants had instructions to rank the statements according to their individual decisions.

### 2.5. Factor Extraction and Interpretation

Factor analysis is used to reduce the number of Q-sets to smaller groups of factors representing a common perspective [46]. The factor analysis was conducted using Ken-Q Analysis v.1.0. After a correlation matrix, a principal component extraction was conducted to organise all of the Q-sort. Factors were rotated using varimax rotation to identify how variables grouped and to maximise the differences between factors.

The process consisted of the following selection criteria for the extraction of factors based on the criteria of Garbellini et al. [36] and their review of the work by Chee et al. [47] and Thompson et al. [48].

1. The starting point was the default number of factors extracted by the Ken-Q analysis software, a total of 8 factors.
2. Factors with an eigenvalue greater than 1.0 were included.
3. At least two significant factor loadings were required for each retained factor.
4. The cross-product of the two highest loadings should be greater than twice the standard error (SE) (Humphrey’s rule). SE was calculated using the formula SE = 1/√n, where n = number of statements in the Q-set. Therefore, loadings of 2 × 1/√40 (factor loadings > 0.31623) identified Q-sorts correlated with each factor.

The interpretation of each factor was performed in two steps: (a) each factor was analysed at a general level; (b) the statements at the two extremes (values of −3, −3, +2, +3) were analysed together to observe the counterpoint of perceptions and compare them to the existing literature.

This comparison enabled the identification of the various perspectives and their influence on the student’s learning, as well as how the result could be usefully extrapolated.

## 3. Results

Questions from n = 37 participants were given, 2 men and 35 women, with ages ranging from 23 to 56 years old ($\bar{x}$ = 34.86; SD = 9.18). Relevant data for the research were also gathered, such as years of experience, the field of expertise, and active participation in colleges or professional associations (Table 2).

The Q-sorts analysis of the 37 participants yielded 8 default factors, focused on 7 viewpoints, according to the selection criteria (Table 3). The selected factors were rotated through varimax rotation to identify variables that could be jointly grouped and maximise the set of the different observations. All the viewpoints included Q-sorts from therapists who worked in different professional fields, with very different durations of their careers, which helps to show the representativity of the viewpoint on the practice of occupational therapy at a global level.

### 3.1. Retained Factor 1: Viewpoint 1—Professional Identity Due to Mentors

The first viewpoint (Table 4) is characterised by the most-valued previous assumptions at the beginning of the occupational therapy training, compared to aspects related to working activity and studies.

The most-valued statement is “I chose my preferred career regardless of other people’s feedback”. With value 2, those that are considered as characteristic of this profile imply aspects linked to concepts or ideas previous to the training. Two of the most significant statements of the factor are linked to previous knowledge sourced from contact with other professionals: “I personally knew some professionals from my future working field”; “My knowledge on the profession of occupational therapist was sourced from a person I knew”. Those aspects related to training seemed to be less relevant in the profile. The least-valued statement, with the greatest statistical strength and representative of this profile is “I think I have spent too long training to become a professional therapist”. Together with the previously mentioned, it can point out a strong professional identity, with previous knowledge of the profession, but not its duties concerning value −2.

### 3.2. Retained Factor 2: Viewpoint 2—A Grey Field on Professional Identity

Viewpoint 2 (Table 4) receives very different influences from the previous one.

This viewpoint shows a clear contrast to the previous one. The most-valued aspect implies the professional experience: “When working on problems in the class, I put myself in the shoes of an occupational therapist professional”. In this regard, many of the arguments of value 2 imply a certain level of poorly defined conditions or situations, such as “I would prefer that occupational therapy had a clearer definition” or “I am still searching for my professional identity”. This indecision is transferred to the least-valued factors or with the highest disagreement, such as “When starting my occupational therapy studies, I had a strong identity of becoming an occupational therapist” or “I was positive I mastered all the required skills to be successful in my career”. In addition to this, in this profile, a vision of professional identities without external mentors emerges, characterised by statements such as “I personally knew some professionals from my future working field” or “I was influenced in my decision to study and become an occupational therapist” in values −2 and −3, respectively.

### 3.3. Retained Factor 3: Viewpoint 3—Reaffirming a Common Professional Identity

Viewpoint 3 (Table 4) has the highest number of Q-sorts included in the analysis, and it is in a unique visible position in extreme values.

The most valued point encompasses situations related to a shared professional identity, where the participant considers himself as a member of a professional group with a positive identification of other occupational therapists through common characteristics with values 2 and 3. At the opposite extreme, with the sharpest disagreement, negative situations for the professional identity can be found, such as “I feel like giving excuses for belonging to this profession”, “I was ashamed to admit I was studying occupational therapy”, or “I still do not know which is my professional identity”.

This coherent comparison between points helps to understand a majority profile of the profession in which the identity feeling is linked to the community or common characteristics among the members of the occupational therapy group.

### 3.4. Retained Factor 4: Viewpoint 4—The Role of Education and Mentors on the Own Professional Identity

Viewpoint 4 (Table 4) includes training and working aspects linked to professional recognition as the most relevant.

The least valued aspects in this profile are those related to aspects previous to the working activity. The only statistically relevant aspect in this point is “I would prefer that occupational therapy had a clearer definition”. Therefore, participants may have a defined or barely ambiguous professional identity. In this case, the explanation could be found in the most-valued counterpoints, which take into account professional relations, both during the training period and the working practice, more specifically, the importance of looking for educators or mentors with whom to identify, for instance, “I admired the occupational therapists/teachers in the areas I thought I was going to work in”, “I feel I share some features with other members of this professional group”, or “When working on problems in the class, I put myself in the shoes of an occupational therapist professional”.

### 3.5. Retained Factor 5: Viewpoint 5—The Outcome of Ongoing Training in Order to Develop a Professional Identity

Viewpoint 5 (Table 4) stands in contrast to the previous ones, based on the lack of mentors.

In this case, the statistically strongest points are placed in two statements related to occupational therapy work and studies (“I think I have spent too long training to become a professional therapist” and “When starting my occupational therapy studies I had a strong identity of becoming an occupational therapist”) against the weak promotion of this role in the classroom (“When working on problems in the class, I put myself in the shoes of an occupational therapist professional”). We believe that the knowledge of the profession has not been sufficiently relevant in this viewpoint and that teaching in the class does not contribute to the understanding of this role. Perhaps the distance between teaching and practice denotes that the lack of knowledge of the role that the participants highlight through characteristics with statistical significance of −2. Regarding role development, we believe, from this point of view, that postgraduate training has been a milestone in the understanding of professional identity, which has enabled the development of an identity held by the participants before their training. It seems that university teaching has not been, in this case, strong enough to develop a professional identity, and further training has been required to shape the concept of professional identity.

## 4. Discussion

Our Q methodology study contributes to a better understanding of the elements that occupational therapists use to construct their professional identity. This examination reveals four dominant viewpoints that shed light on the different ways in which occupational therapists identify with their profession. Our study emphasises the importance that therapists place on the three stages that structure the various perspectives of their professional identity: university training, the period before university training, and the period after university training.

### 4.1. Before University Training

The findings showed that Viewpoint 1 (professional identity, thanks to mentors) gives a clear image of a vocational discipline. Given that professional identity encompasses professional self-perception [49], it is evident how participants join a discipline with some knowledge of its professional goals and skills. Monrouxe [50] and Vignoles et al. [51] have argued that many factors contribute to the professional identity of a health professional, including social class, ethnic origin, personal values and beliefs, their perception of being unique, and the interpretation of others’ perceptions [52]. Our study confirms these factors, along with the influence of five viewpoints:

(a) Personal: Mark et al. [52] described how personal relationships, social class, previous education, and social environmental factors can shape an individual’s professional identity. As a result of the multiplicity and interaction of these factors, it has been suggested that professional identity, even before starting university studies, follows its own path, which is determined by the person who chooses to study occupational therapy over other disciplines [50,53].
(b) Family: As with other disciplines, such as nursing [52], studies included in the research by Mao et al. [54] suggest that family support is closely linked to the selection of a particular degree. Our study confirms that nursing students who have relatives working in the health sector express a higher interest in completing a certain training program [55,56,57].
(c) Professional: Mark et al. [52] noted that clinical experience, role models (e.g., teaching staff, preceptors, and mentors), and exposure to the profession before formal training influence the establishment of a professional identity [5,50,53]. Rituals, rites of passage, and symbols in education and health institutions can also contribute to the creation of a professional identity [50].
(d) Others: As in the study by Mark et al. [52], the interest in pursuing occupational therapy studies is linked to an individual’s exposure to a professional before professional training [5] or to the discourse held by the institutions where the studies were completed.
(e) Gender: Finally, we believe that gender is a core element in the decision to study occupational therapy. Our sample corroborates that only 8% of occupational therapists are men, while 92% are women [58], and 10% are LGBT [59]. This situation is similar to that in other disciplines. Although occupational therapy has traditionally been considered a female-dominated occupation, it is experiencing an increase in the number of male professionals. However, this number is insufficient to achieve professional parity, contributing to the gender inequality present in most professions [60].

### 4.2. During University Training

This section of the study explored the influence of the academic and clinical practice environments on the establishment of professional identity among occupational therapy students. The findings from Viewpoint 2 (a grey field on professional identity) offered mixed views on the impact of university training on the development of professional identity. The study revealed that the concept of professional identity was not adequately structured during the university training stage, leading to a crisis among students. As a result, participants compensated for the lack of training by seeking mentors who could serve as role models. This was highlighted in Viewpoint 4 (the role of education and mentors on the own professional identity).

In Viewpoint 2, the study found that the level of professional identity decreased as students progressed in their professional studies. This finding was consistent with other disciplines, such as nursing [54]. The increase in professional skills and knowledge did not strengthen students’ confidence to provide care. The authors agreed with Khodaei et al. [61] and Shaterjalali et al. [62], in that the disconnection between theoretical and practical training may contribute to a decrease in professional identity. This may occur because theory trainers tend to teach ideal knowledge, while practical trainers provide information regarding real activities.

The lack of a clear professional identity was identified as a crisis point among participants. While well-managed crises can contribute to personal and professional growth, an insufficient feedback mechanism may worsen the crisis [63,64]. Participants in the study felt the need to justify their work, in comparison to other socially better-considered occupations, such as medicine, nursing, and psychology. This scenario was previously described by Mark et al. [52], who emphasised that other professionals considered biomedical models and psychological theories better than professional activities. This situation led to occupational therapists being questioned about the value of their profession and the justification of their practices based on their occupation.

The transition from a student to a professional therapist was stressful for participants, as highlighted by Pillen et al. [65] and Volkmann and Anderson [66]. The authors noted that this stage is frequently described as a fight that can be triggered by relational dilemmas, which can threaten a student’s professional identity. When students are allowed to articulate their thoughts on their skills, experiences, and needs, a space for the knowledge and growth of their professional identity is provided to them [67,68,69,70,71,72]. This reflection, as quoted by Pang [73], helps to reciprocally consolidate confidence, competency, and professional identity.

The authors agreed with Ibarra [74] and Goldie [75] that university training enables a high number of theoretical spaces. However, it did not help participants, in this particular case, to identify who they are and whom they want to become in their professional role, in terms of their attributes, beliefs, values, reasons, and experiences. This situation affects the development of the occupational therapist’s professional identity, since the concept of feeling, thinking, and acting as an occupational therapist is not internalised [76].

The lack of a clear professional identity led to role confusion, mainly due to others’ perceptions of the occupational therapy profession, as well as the perceptions of the professionals of their own work. This role confusion generated an understanding of the discipline both from the inside and the outside, as warned by Ashby et al. [63], Drolet and Desormeaux-Moreau [17], Edwards and Dirette [42], Sauvageau et al. [77], and Turner and Knight [26]. The study found that this confusion impacted the way occupational therapists carried out their job, increasing the level of emotional anguish, generating a lack of interest, and promoting stereotypes of the discipline.

In Viewpoint 4, participants emphasised the important role of mentors and supervisors in the development of their professional identity during their practical training. The influence of role models on the development of professional identity has been previously documented in the literature. For example, Mark et al. [52] described the importance of interaction with referent professionals in the discipline for transferring knowledge and skills, which, in turn, can contribute to the development of professional identity.

Our study findings support the idea that the image projected by mentors and supervisors was assimilated by participants and became a key factor in the development of their professional identity. Participants highlighted the importance of these relationships in shaping their understanding of the core values and beliefs of the occupational therapy profession, which played a significant role in the development of their own professional identity.

The concept of professional identity development can be understood in the context of the four stages described by Sauvageau et al. [77]: crisis, awakening, exploration, and commitment. The practical training stage, where participants had the opportunity to work with mentors and supervisors, appears to have been an awakening stage for our participants. This is supported by the emergence of a sense of direction and purpose in their professional practice, as well as a greater level of confidence in their abilities and professional identity.

Overall, our study highlights [78,79,80,81] the important role that mentors and supervisors can play in the development of professional identity among occupational therapy students. It also sheds light on the challenges that students face in developing a strong professional identity during their university training [82,83,84,85]. As such, these findings have important implications for the design of educational programs and training models for occupational therapy students, with a particular focus on the role of mentors and practical training experiences in supporting the development of a strong and resilient professional identity [86,87].

### 4.3. After University Training

Our findings regarding the professional identity of occupational therapists, highlighted in Viewpoint 3 (reaffirming a common professional identity), show a need to share working spaces with other occupational therapists, in order to consolidate and develop the professional identity. Even once they are in the labour market, occupational therapists keep putting their trust in training, as can be seen in Viewpoint 5 (the outcome of ongoing training to develop the professional identity), to complement university training with more benefits than drawbacks [49].

Professional identity can become even more specific when taking working experience into account [88]. Understanding that professional responsibility implies learning the patient’s needs will open the door to understanding what being an occupational therapist means [82]. For this reason, the transition to practice for recently graduated occupational therapists may be challenging [70,72,89]. Moores and Fitzgerald [90] mention that new graduates face many demands, including those made by complex working environments [91,92] and those created by a discrepancy between their expectations and their hands-on experience.

We agree with the participants in our study that interaction with other occupational therapists promotes professional identity, since the latter develops from a sense of belonging and uniqueness [49]. Professional identity is greatly reinforced by non-clinical work, such as teaching or research together with other colleagues, and it is essential to any discipline where students or recent graduates are barely aware of it [75,90,93]. Seeking support from old supervisors and colleagues was an action that led to their practice, which became a clear and relevant model to reaffirm their professional identity. In relation to this, Moores and Fitzgerald [90] not only mentioned this cooperation between professionals of the same discipline, but also highlighted that team members and colleagues may become a relevant resource to help recent graduates to undergo their transition.

From our point of view, as stated previously in the study, the evolution of professional identity also takes place thanks to interactions with others. We agree with Goldie [75] and Warmington and McColl [93] that the development process of professional identity encompasses a transformation period of mainly a social and relational nature. Through this relationship with their peers, occupational therapists are exposed to their discourse and update norms, values, and roles of their profession, which the therapist can compare with and differentiate from [94,95].

A poor transition in the identity-forging process may entail a professional crisis. Participants mention them in this study from the approach of a lack of confidence in their actions and decision-making process. These aspects have already been examined by Holland et al. [67], who raised the alarm about the fact that recently graduated students expressed their need to feel confident in their role [70], since after six months, a professional confidence crisis arises. We put this crisis down to a problematic transition from a student to a professional, which is mainly due to a lack of understanding, belief in their role, awareness of the limits in their discipline, and deprecation of the profession, all this influenced by the nonachievement of those expectations, which leads them to a lack of confidence, as mentioned by Holland et al. [67].

Regarding this, the latency of this crisis may linger until the first years of working experience, which is confirmed by this study. The difference changes in the role during the first years and the transition over periods of one or two years to gain professional expertise and to adapt to the complexity of the working environment make participants more familiar with the daily routine and prove to be more competent. As happens in other disciplines, such as nursing [54], we believe that professional identity starts to outline after the first five years.

In our opinion, the initial crisis faced by occupational therapists can be attributed to the demands of a labour world where interprofessional cooperation generates complex challenges that some professionals struggle to solve [2]. Furthermore, other professions with a strong professional identity require decision-making or problem-solving that cannot be achieved by many occupational therapists. We concur with Best and William [49] that interdisciplinary teamwork poses a challenge for occupational therapists.

Professional identity is constantly evolving, and ongoing training is one of the defining features of the occupational therapy profession, as it helps to acknowledge the remaining attributes that contribute to our understanding of professional identity [82]. Knowledge drawn from ongoing training enhances the confidence of professionals in their work by providing expertise [67]. As professionals grow, their need to specialise in their training also grows. We believe that ongoing training strengthens professionals’ confidence in their tasks by providing expertise [67].

We believe that university training may leave some gaps in participants’ knowledge and that efficient ongoing training is essential to overcoming professional technicisms and adapting knowledge to the professional reality [96]. Ongoing training managed by professional colleges and associations [97], or even university postgraduate courses, allows individuals to have more autonomy in choosing the type of training they would like to undergo, in contrast to a rigid university training that does not always match the working practice. We think that ongoing training should be focused on understanding the skills required for the occupational therapist profession by identifying the main features of the professional profile in the working practice.

We have observed that participants emphasise the importance of ongoing education over undergraduate education, as occupational therapists realise that ongoing training is not a passive experience [98]. Ongoing training helps professionals find answers to the development of the discipline, establish connections between concepts learned during the undergraduate period and new, context-specific ones, and gain experience. Under these circumstances, all that has been learned merges with new knowledge, experience, and confidence and reflects ability, yielding professional growth. Benner [99] and Mao et al. [54] suggested five stages that all professionals must pass through in the development of their professional identity after training. These five stages (beginner, advanced beginner, competent, professional, and expert) are also proposed by Schell and Schell [100] or Talavera [41], in the case of the development of professional reasoning skills for occupational therapists. This leads us to believe that professional reasoning and professional identity are linked to professional growth, as highlighted by Moruno [101], Schell and Schell [100], and Talavera [41].

### 4.4. Limitations and Strengths of the Study

The fact that participants were recruited from different working fields could be considered the main sampling potential limitation. Nevertheless, these differences were monitored and were taken into account to optimise the group’s heterogeneity to obtain the greatest diversity of viewpoints. Another potential limitation could be related to the whole sample, since these are occupational therapists exclusively located in Spain, which may hinder the ability to transfer the outcome to professionals in other contexts.

Despite this, the aim of Q methodology, and our study, is to display the viewpoint and opinion diversity rather than favouring the transfer of this outcome. The strengths of this study lie in its potential transferability and its use of Q methodology. Our findings on Spanish occupational therapists’ professional identity can serve as a framework for future studies in this area. Q methodology’s focus on subjectivity may uncover diverse perspectives, thus enabling the framework to expand. Additionally, these findings may support the development of stronger professional identities and improved professionals.

### 4.5. Future Lines

In this field, and with the potential of Q methodology: (a) A comprehensive study on the educators’ training could be undertaken, since they impact in one way or another the professional identity of the participants; (b) it could be channelled to stages prior to university entry; (c) the influence of working environments where occupational therapists carry out their internships could also be analysed.

## 5. Conclusions

The findings of this study lead us to the conclusion that occupational therapy is a vocation, but there are gaps in understanding the tasks of occupational therapists at the university stage, which hinders the development of a professional identity. The study suggests that university training alone may not be sufficient to build a solid understanding of professional identity, and internships with external mentors during the university stage may be necessary for its consolidation. As occupational therapists work with other professionals, there must be enough discussion to encourage professional identity, while undergoing more discipline-oriented training as the years go by.

This study contributes to a broader understanding of the work culture in occupational therapy by questioning some aspects of university training that the participants mentioned concerning the forging of professional identity. The results provide a view of the priorities shared by the occupational therapists who took part in this research. This awareness may not only impact the understanding of the professional identity formation process, but also provide a solid context for supporting occupational therapists in developing their professional identity.

It can be concluded that there is no book with a universal answer on how to develop a professional identity. However, this study confirms that decision-making, confidence, relationships with colleagues from the same or different disciplines, reasoning, mentorship, practical experience, ongoing training, and the ability to adapt to changes are core factors of professional identity for the participants in this study.

## Figures and Tables

**Table 1:** Q sample statements prepared for Q-sort.

No.	Statement
**Group 1. Longitudinal statements**	
1	I think professional identity is a relevant factor during the training for professional practice.
2	I think occupational therapy is too diverse to have a distinctive professional identity
3	I would prefer that occupational therapy had a clearer definition
4	I feel confident when describing occupational therapy
5	I consider that overcoming challenging situations and/or experiences fosters professional growth
6	Working as an occupational therapist makes me happy
7	I have developed a clear professional identity
8	I am still searching for my professional identity
9	I know whom I am professionally speaking
10	I still do not know which is my professional identity
**Group 2. Influences before occupational therapy education**	
11	I knew the nature of the tasks performed in my future career
12	In most working environments, professionals from different disciplines work together
13	I personally knew some professionals from my future working field
14	I followed the updates regarding my professional field in newspapers, TV, social media, events, etc.
15	My decision to study and become an occupational therapist was influenced
16	I chose my preferred career regardless of other people’s feedback
17	My knowledge of the profession of occupational therapy was sourced from a person I knew
18	I considered I should take into account both my own ideal and my surrounding factors during the election process of my career
19	I was sure I was successful in occupational therapy
20	I knew the different skills of the professional categories with which I was going to cooperate
**Group 3. Influences during occupational therapy studies**	
21	I knew what type of therapies were going to be part of my working activity
22	I knew what type of environments would be part of my working activity
23	I knew what type of tasks would be part of my working activity
24	When working on problems in class, I put myself in the shoes of an occupational therapist professional
25	During my last years of training, I already applied the professional reasoning of an occupational therapist
26	I admired the occupational therapists/educators in the areas I thought I was going to work in
27	I was confident I could carry out an excellent job in occupational therapy
28	I was positive I mastered all the required skills to be successful in my career
29	When starting my occupational therapy studies, I had a strong identity of becoming an occupational therapist
30	I was ashamed to admit I was studying occupational therapy
**Group 4. Influences while working**	
31	I do not feel sufficiently trained to work as an occupational therapist
32	I think I will easily perform my tasks as a professional therapist
33	I like working as a professional therapist
34	I think I have spent too long training to become a professional therapist
35	I try to learn about the scene of another professional field to strengthen my professional belief
36	I feel a member of this professional group
37	I can positively identify with other occupational therapists
38	I feel I share some features with other members of this professional group
39	I feel like giving excuses for belonging to this profession
40	I take advantage of my skills as an occupational therapist

**Table 2:** Participant variables and characteristics.

Q-Sort	Age	Genre	YEX	APOT	Field of Expertise
2	33	Male	11	0	Pediatrics
9	33	Female	11	5	Pediatrics
13	26	Female	1	2	Pediatrics
29	23	Female	1	0	Geriatrics
6	24	Female	1	1	Neurorrehabilitation
7	29	Female	6	1	Community
11	39	Female	18	17	Various
25	30	Female	9	0	Geriatrics
30	26	Female	4	0	Geriatrics
34	35	Male	10	5	Geriatrics
4	23	Female	0	1	Neurorrehabilitation
5	40	Female	18	15	Geriatrics
8	25	Female	3	0	Geriatrics
10	31	Female	8	5	Mental health
18	40	Female	18	2	Geriatrics
19	53	Female	17	18	Geriatrics
22	38	Female	16	4	Various
23	23	Female	0	0	Geriatrics
26	37	Female	16	0	Various
27	37	Female	16	16	Mental health
28	24	Female	1	2	Geriatrics
31	30	Female	8	9	Mental health
33	38	Female	18	0	Mental health
3	56	Female	29	30	Mental health
15	24	Female	2	3	Various
17	38	Female	17	2	Geriatrics
32	39	Female	13	13	Mental health
35	44	Female	18	18	Geriatrics
36	50	Female	25	0	Various
37	29	Female	7	5	Various
20	35	Female	12	11	Geriatrics
24	42	Female	20	11	Various
1	53	Female	3	1	Geriatrics
14	45	Female	6	6	Various
12	26	Female	3	4	Pediatrics
21	37	Female	13	2	Geriatrics

YEX: Years of experience; APOT: Active participation in OT associations (years).

**Table 3:** Application of selection criteria.

Selection criteria								
Default factors	1	2	3	4	5	6	7	8
Eigenvalue (value needed >1.0)	15.31	3.20	2.19	1.95	1.63	1.38	1.26	1.16
% explained variance	41	9	6	5	4	4	3	3
Number of factor loading > 0.31623	4	6	13	7	2	2	1	2
Humphrey’s rule succeeded	Y	Y	Y	Y	Y	Y	N	Y
More than 2 factors with *p* < 0.05	Y	Y	Y	Y	Y	N	-	N

Y = Yes; N = No.

**Table 4:** Complete list of 40 Q-sort statements and idealised sorts for the five patterns.

	Statement	V1	V2	V3	V4	V5
1	I think professional identity is a relevant factor during the training for professional practice.	+2	+2	+2	+2	+2
2	I think occupational therapy is too diverse to have a distinctive professional identity	0	+1	0	−1 ^b^	+1
3	I would prefer that occupational therapy had a clearer definition	+2	+2	+1	−2 ^a^	+2
4	I feel confident when describing occupational therapy	+1	0	+1	+1	0
5	I consider that overcoming challenging situations and/or experiences fosters professional growth	+1	+2	+2	+1	+2
6	Working as an occupational therapist makes me happy	0	0	+1 ^e^	+1	0
7	I have developed a clear professional identity	0	−2 ^b^	+1	0	0
8	I am still searching for my professional identity	+1	+2	0	−1 ^b^	+1
9	I know whom I am professionally speaking	−1	0	+1	0	+2
10	I still do not know which is my professional identity	−1	+1	−2	−1	0
11	I knew the nature of the tasks performed in my future career	−2 ^b^	0	0	0	−1
12	In most working environments, professionals from different disciplines work together	−1 ^a^	+2	+1 ^c^	+1	+2
13	I personally knew some professionals from my future working field	+2 ^d^	−2	−1	−2	−2
14	I followed the updates regarding my professional field in newspapers, TV, social media, events, etc.	+1	−1	−2	−2	0
15	My decision to study and become an occupational therapist was influenced	+1 ^c^	−3	−2	−2	1 ^d^
16	I chose my preferred career regardless of other people’s feedback	+3	+1	0	+3	0
17	My knowledge of the profession of occupational therapy was sourced from a person I knew	+2 ^d^	−2	−1	−2	−2
18	I considered I should take into account both my own ideal and my surrounding factors during the election process of my career	−2	+1	0	0	−1
19	I was sure I was successful in occupational therapy	+2 ^d^	0	0	−1	0
20	I knew the different skills of the professional categories with which I was going to cooperate	−1 ^a^	−1	0	−1	0
21	I knew what type of therapies were going to be part of my working activity	−2	−1	−1	0	−1
22	I knew what type of environments would be part of my working activity	−2	−1	−1	+1 ^d^	−1
23	I knew what type of tasks would be part of my working activity	−1	0	0	0	−2
24	When working on problems in class, I put myself in the shoes of an occupational therapist professional	+1	+3 ^e^	−1 ^c^	+2	−2 ^a^
25	During my last years of training, I already applied the professional reasoning of an occupational therapist	−1	0	−2	0	−1
26	I admired the occupational therapists/educators in the areas I thought I was going to work in	+1	0	−1	+2	0
27	I was confident I could carry out an excellent job in occupational therapy	0	−1	0	+1	0
28	I was positive I mastered all the required skills to be successful in my career	0	−2	−1	+1	0
29	When starting my occupational therapy studies, I had a strong identity of becoming an occupational therapist	0	−2 ^a^	−1 ^f^	0	+2 ^d^
30	I was ashamed to admit I was studying occupational therapy	0 ^d^	−2	−2	−3	−2
31	I do not feel sufficiently trained to work as an occupational therapist	0	+1	−2	−1	−2
32	I think I will easily perform my tasks as a professional therapist	−1	0	0	0	+1
33	I like working as a professional therapist	+1	+1	+2	+2	+1
34	I think I have spent too long training to become a professional therapist	−3 ^a^	0	1	−1 ^c^	+3 ^d^
35	I try to learn about the scene of another professional field to strengthen my professional belief	−2	+1	+1	−1	−1
36	I feel a member of this professional group	+2	−1	+3	+2	−1
37	I can positively identify with other occupational therapists	+1	+1	+2 ^e^	+1	−1
38	I feel I share some features with other members of this professional group	0	+1	+2	+2	+1
39	I feel like giving excuses for belonging to this profession	−2	−1 ^e^	−3	−2	−3
40	I take advantage of my skills as an occupational therapist	0	−1	+2	0	+1

V1: Viewpoint 1—Professional identity, thanks to mentors; V2: Viewpoint 2—A grey field on professional identity; V3: Viewpoint 3—Reaffirming a common professional identity; V4: Viewpoint 4—The role of education and mentors on the own professional identity; V5: Viewpoint 5—The outcome of ongoing training in order to develop a professional identity; The numbers ranging from −3 to +3 correspond to location of the statements in an idealized each pattern, placed on a grid, as is shown in Figure 1. Note: a = **◄; b = *◄; c: *; d = ►**; e = ►*; f: **.

## Data Availability

The author confirms that all data generated or analysed during this study are included in this published article. Furthermore, primary and secondary sources and data supporting the findings of this study were all publicly available at the time of submission.
